# Supplementary material for: CO2-Switchable Hierarchically Porous Zirconium-Based MOF-Stabilized Pickering Emulsions for Recyclable Efficient Interfacial Catalysis
Source: Materials (Basel). 2023 Feb 17;16(4):1675. doi: 10.3390/ma16041675 (PMC9960431; doi:10.3390/ma16041675)
Supplement: Supplementary file 1 [file materials-16-01675-s001.zip › materials-2151229-supplementary.pdf]

Supporting Information

CO_2-Switchable Hierarchically Porous Zirconium-Based MOF-Stabilized Pickering Emulsions for Recyclable Efficient Interfacial Catalysis

Xiaoyan Pei*, Jiang Liu, Wangyue Song, Dongli Xu, Zhe Wang and Yanping Xie

College of Chemistry and Chemical Engineering, Xinyang Normal University, Xinyang 464000, China.

* Correspondence: xiaoyanpei2009@163.com

Number of pages: 16

Number of figures: 28

Number of tables: 3

Table of Contents

1. Experimental section…………………………………………………………..…..S3
2. Figures S1-S28……………………………………………………….….…..…... S4
3. Tables S1-S3…...……………………………………………………….……........ S16

1. Experimental section

Materials

$ZrCl_4$, $Zn(NO)_3{\cdot}6H_2O$, benzoic acid, 2,5-dihydroxyterephtalic acid, and 2,5-dihydroxyterephthalic acid were obtained from Aladdin. (3-Aminopropyl)trimethoxysilane (APTMS), 3-(2-aminoethylamino)propyltrimethoxysilane (AEAPTMS) and 3-[2-(2-aminoethylamino)ethylamino]propyl-trimethoxysilane (AEAEAPTMS) were obtained from Energy Chemical Co., Ltd. CO_2 (Praxair, SFC grade, 99.998 vol%) and N_2 (Praxair, 99.9993 vol%) were obtained from Praxair. All the chemicals were used as received unless otherwise stated.

Instrumentation

X-ray photoelectron spectroscopy (XPS) were determined on a Thermo Scientific K-Alpha electron energy spectrometer using Al Kα (1486.6 eV) as the X-ray source. X-ray diffraction (XRD) patterns were measured on a Rigaku SmartLab9 diffractometer with monochromatic Cu Kα radiation (λ = 1.5418 Å). Scanning electron microscopy (SEM) were recorded on a Hitachi S-4800 microscope at 5 kV. Nuclear magnetic resonance (NMR) spectra were carried out on a JNM-ECZ600R/S3 spectrometer (600 MHz). Mass spectra were measured on an Agilent GC-MS-5890A/5975C Plus spectrometer (EI). Water contact angles were obtained through a KRÜSS Drop Shape Analyzer 25 (KRÜSS DSA25).

2. Figures S1-S28

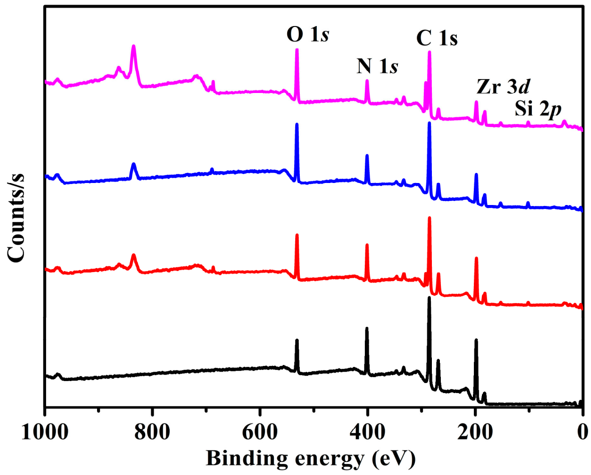

Figure S1 XPS spectrum of (a) H-UiO-66-$(OH)_2$, (b) H-UiO-66-$(OAPTMS)_2$, (c) H-UiO-66-$(OAEAPTMS)_2$ and (d) H-UiO-66-$(OAEAEAPTMS)_2$.

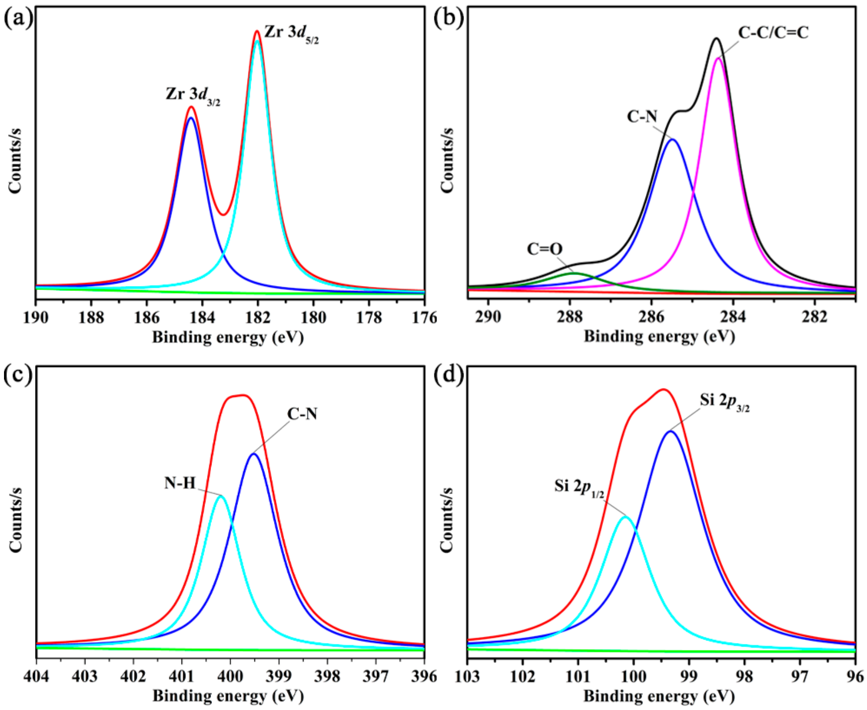

Figure S2 High-resolution XPS spectrum of Zr 3*d* (a), C 1*s* (b), N 1*s* (c) and Si 2*p* (d) of H-UiO-66-$(OAPTMS)_2$.

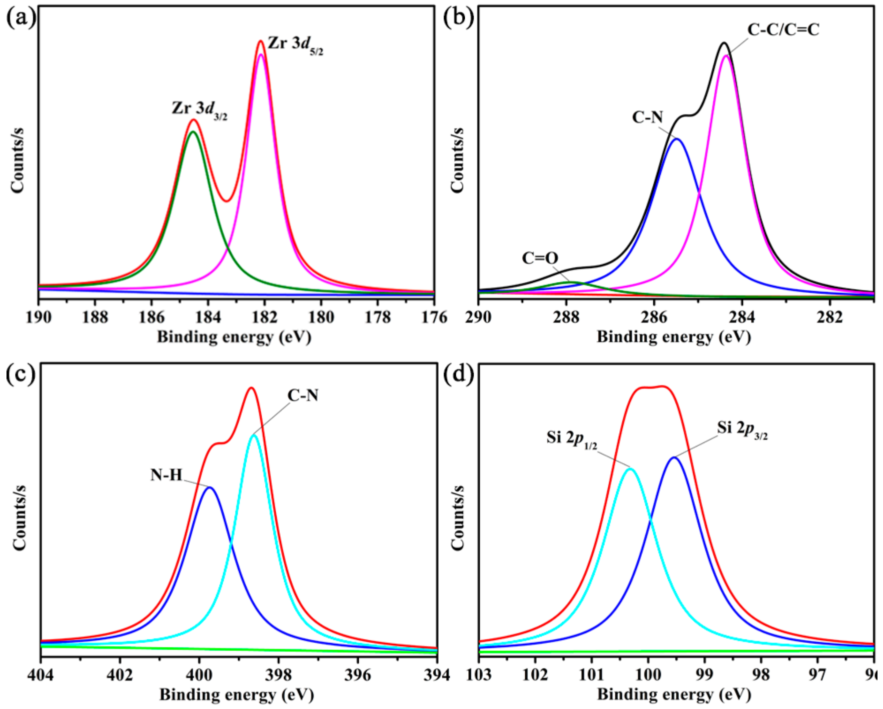

Figure S3 High-resolution XPS spectrum of Zr 3*d* (a), C 1*s* (b), N 1*s* (c) and Si 2*p* (d) of H-UiO-66-$(OAEAPTMS)_2$.

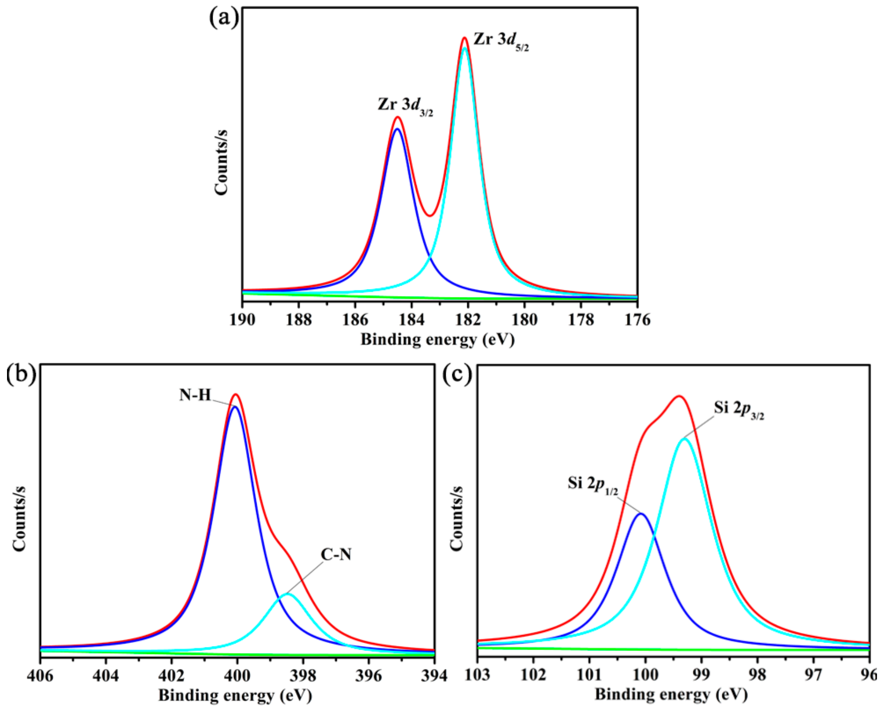

Figure S4 High-resolution XPS spectrum of Zr 3*d* (a), N 1*s* (b) and Si 2*p* (c) of H-UiO-66-$(OAEAEAPTMS)_2$.

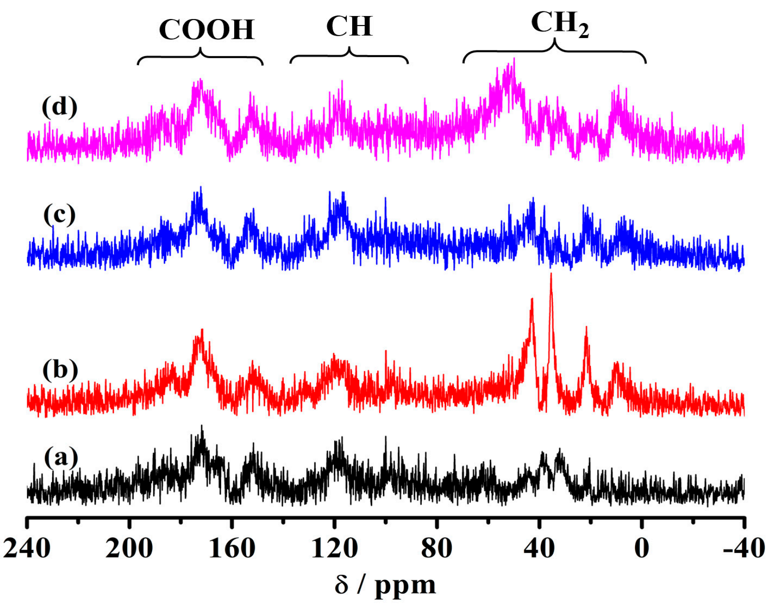

Figure S5 Solid-state ^{13}C NMR spectra of (a) H-UiO-66-$(OH)_2$, (b) H-UiO-66-$(OAPTMS)_2$, (c) H-UiO-66-$(OAEAPTMS)_2$ and (d) H-UiO-66-$(OAEAEAPTMS)_2$.

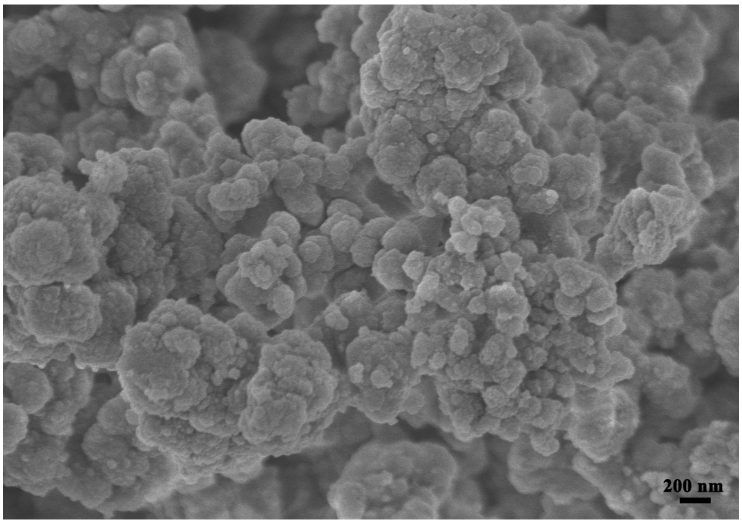

Figure S6 SEM image of the pristine H-UiO-66-$(OH)_2$.

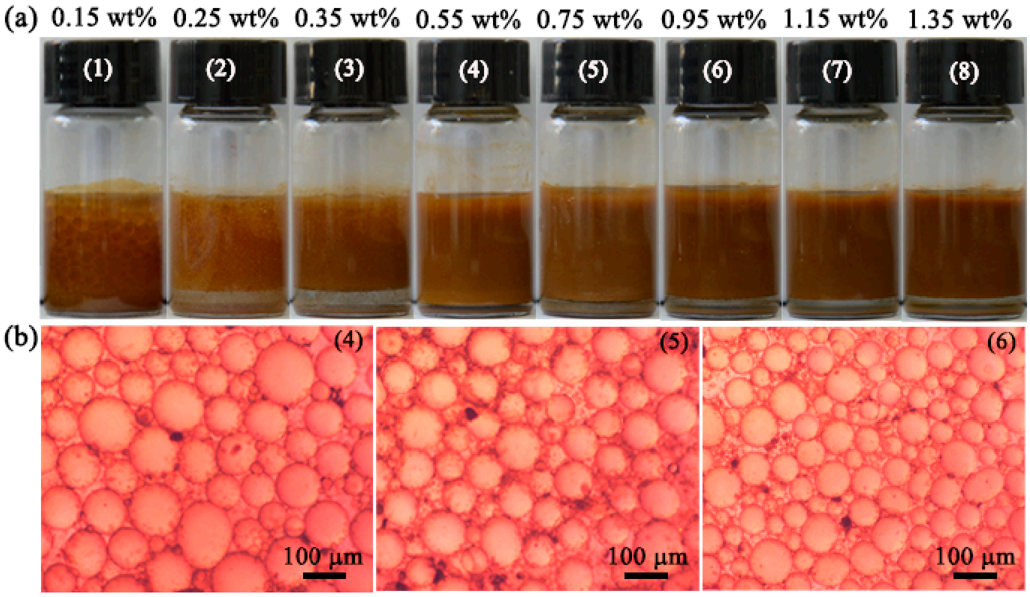

Figure S7 Photographs of toluene-in-water emulsions stabilized by H-UiO-66-(OAPTMS)$_2$ (a) and chosen micrographs for (4), (5) and (6) in (a) (b).

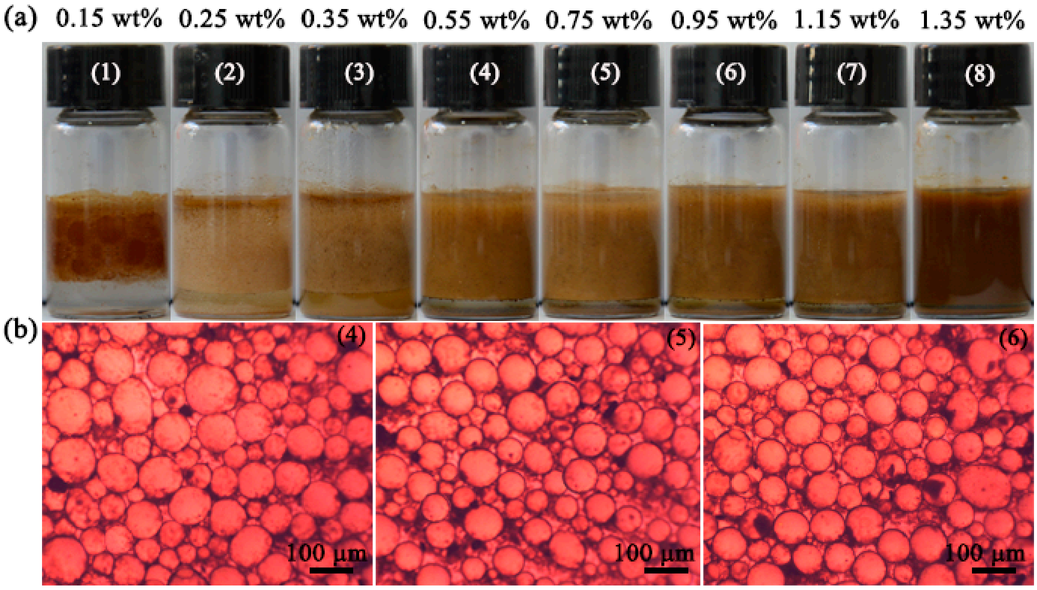

Figure S8 Photographs of toluene-in-water emulsions stabilized by H-UiO-66-(OAEAPTMS)$_2$ (a) and chosen micrographs for (4), (5) and (6) in (a) (b).

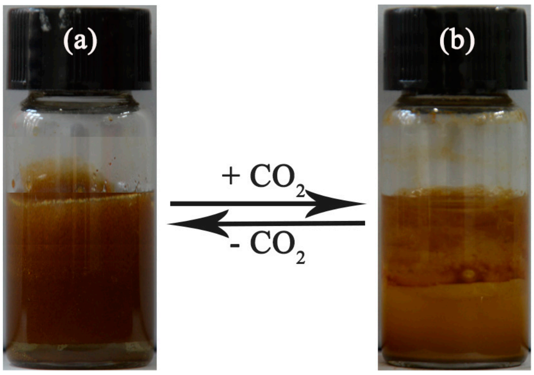

Figure S9 Photographs of benzene (3 ml)-water (2 ml) emulsion stabilized by H-UiO-66-$(OAPTMS)_2$ (0.55 wt%): (a), before CO_2 bubbling; (b), after CO_2 bubbling.

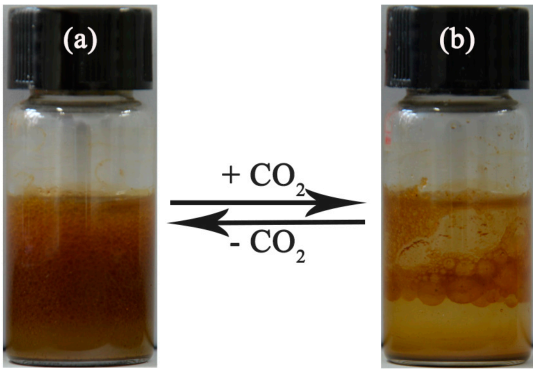

Figure S10 Photographs of benzene (3 ml)-water (2 ml) emulsion stabilized by H-UiO-66-$(OAEAPTMS)_2$ (0.55 wt%): (a), before CO_2 bubbling; (b), after CO_2 bubbling.

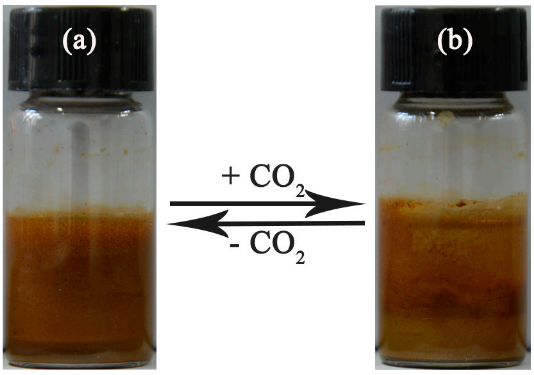

Figure S11 Photographs of benzene (3 ml)-water (2 ml) emulsion stabilized by H-UiO-66-$(OAEAEAPTMS)_2$ (0.55 wt%): (a), before CO_2 bubbling; (b), after CO_2 bubbling.

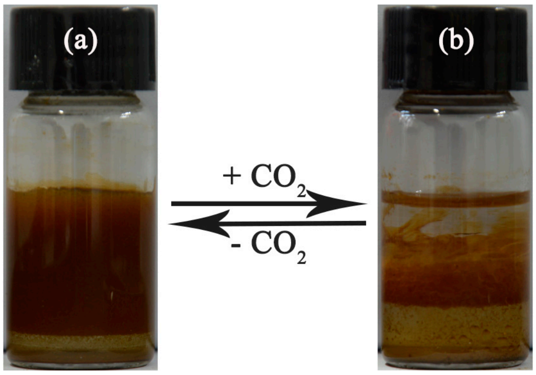

Figure S12 Photographs of n-hexane (3 ml)-water (2 ml) emulsion stabilized by H-UiO-66-(OAPTMS)$_2$ (0.55 wt%): (a), before CO_2 bubbling; (b), after CO_2 bubbling.

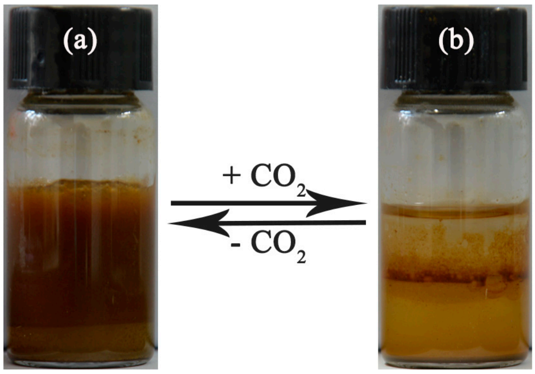

Figure S13 Photographs of n-hexane (3 ml)-water (2 ml) emulsion stabilized by H-UiO-66-(OAEAPTMS)$_2$ (0.55 wt%): (a), before CO_2 bubbling; (b), after CO_2 bubbling.

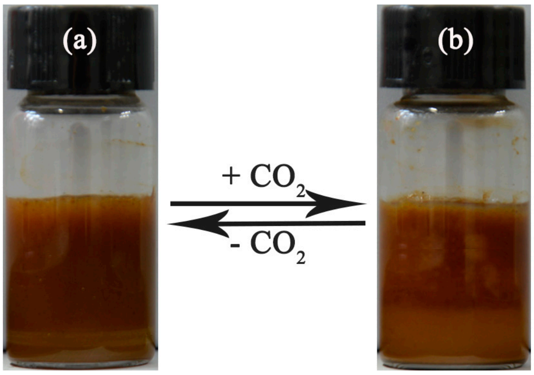

Figure S14 Photographs of n-hexane (3 ml)-water (2 ml) emulsion stabilized by H-UiO-66-(OAEAEAPTMS)$_2$ (0.55 wt%): (a), before CO_2 bubbling; (b), after CO_2 bubbling.

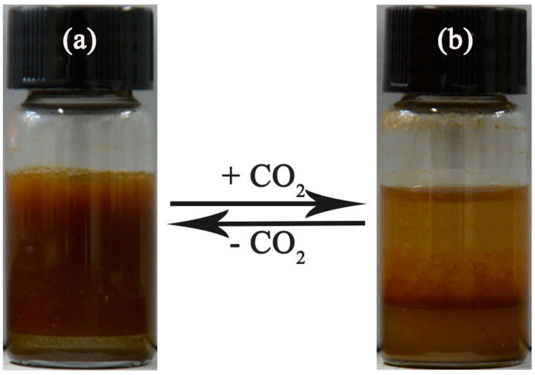

Figure S15 Photographs of cyclohexane (3 ml)-water (2 ml) emulsion stabilized by H-UiO-66-$(OAPTMS)_2$ (0.55 wt%): (a), before CO_2 bubbling; (b), after CO_2 bubbling.

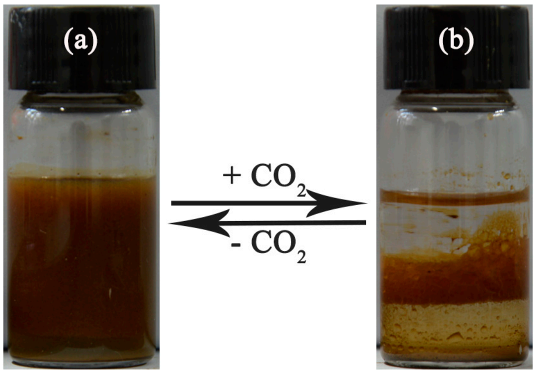

Figure S16 Photographs of cyclohexane (3 ml)-water (2 ml) emulsion stabilized by H-UiO-66-$(OAEAPTMS)_2$ (0.55 wt%): (a), before CO_2 bubbling; (b), after CO_2 bubbling.

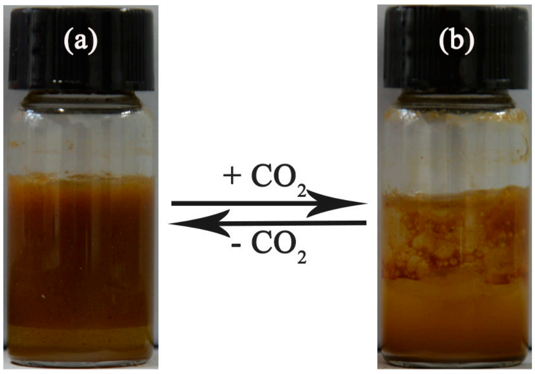

Figure S17 Photographs of cyclohexane (3 ml)-water (2 ml) emulsion stabilized by H-UiO-66-$(OAEAEAPTMS)_2$ (0.55 wt%): (a), before CO_2 bubbling; (b), after CO_2 bubbling.

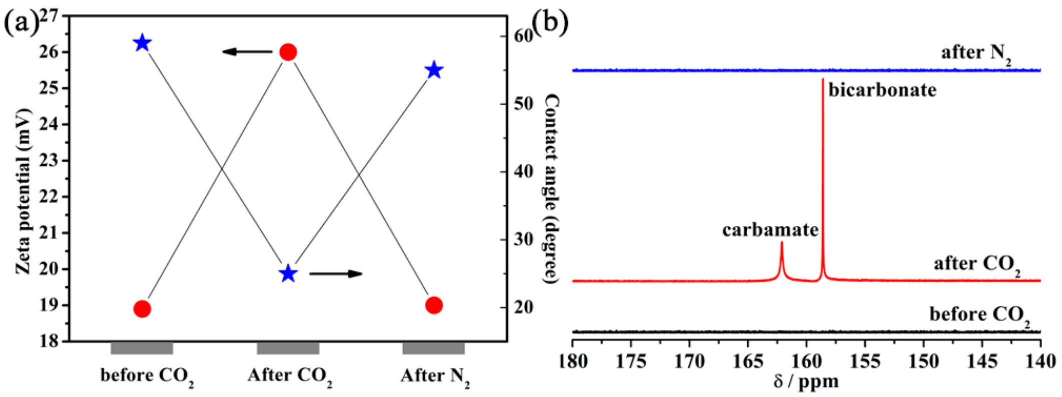

Figure S18 Zeta potential and contact angle of H-UiO-66-(OAPTMS)2 before CO_2, after CO_2 and after N_2.

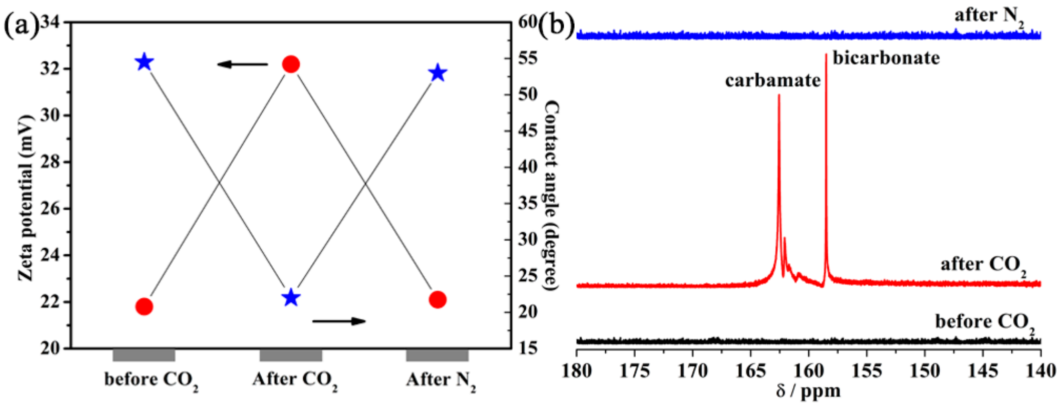

Figure S19 Zeta potential and contact angle of H-UiO-66-(OAEAPTMS)2 before CO_2, after CO_2 and after N_2.

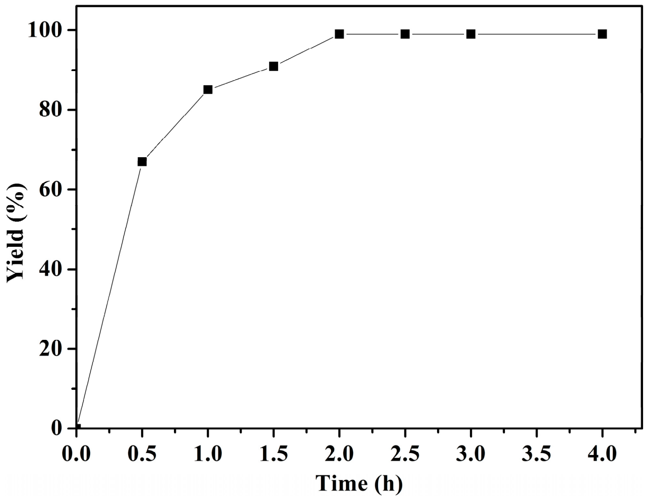

Figure S20 The variation of 2-benzylidenemalononitrile yield with reaction time in H-UiO-66-(OAEAEAPTMS)2-based Pickering emulsion at 25°C

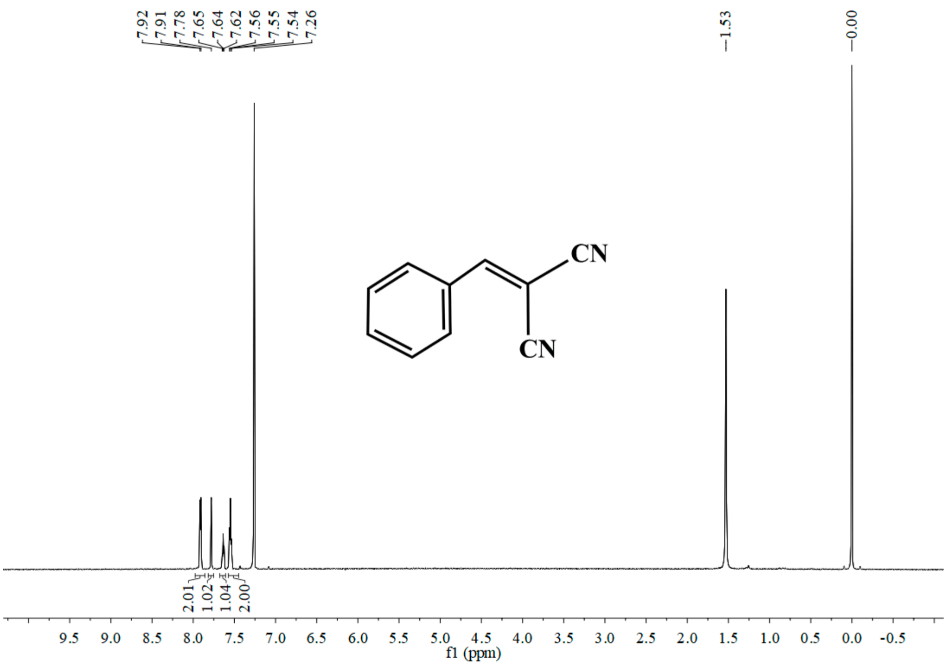

Figure S21 ^{1}H NMR of 2-benzylidenemalononitrile.

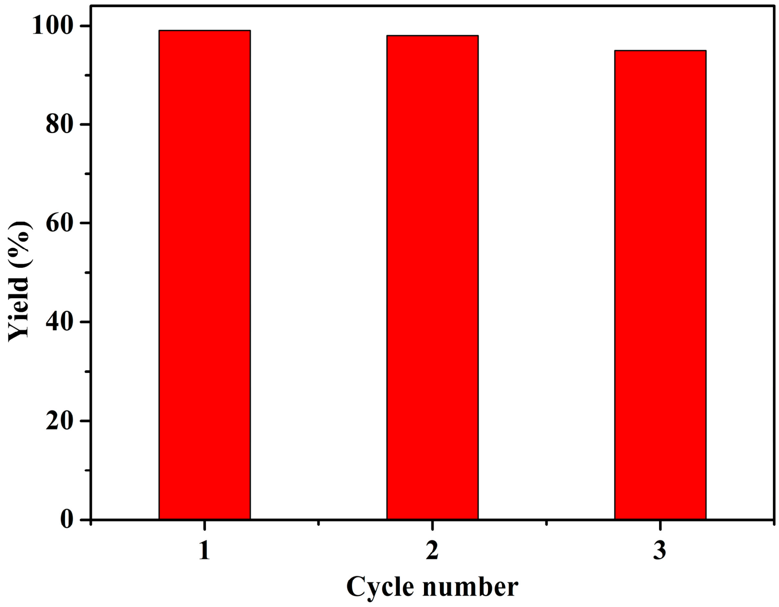

Figure S22 The recyclability of Pickering emulsion in the reaction of benzaldehyde with malononitrile.

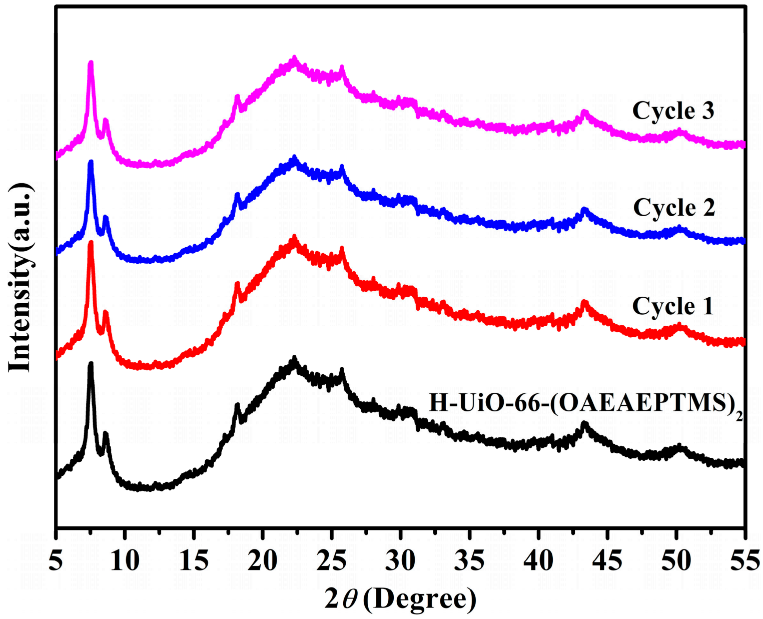

Figure S23 PXRD patterns of H-UiO-66-$(OAEAEPTMS)_2$ before and after each catalytic run.

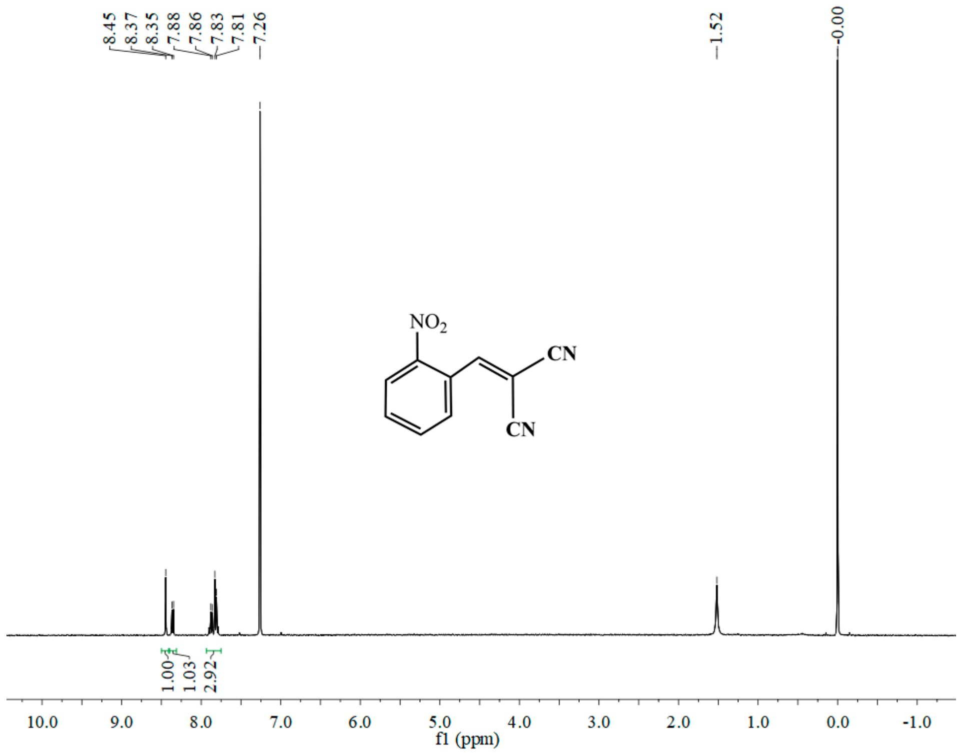

Figure S24 ^{1}H NMR of 2-(2-nitrobenzylidene)malononitrile.

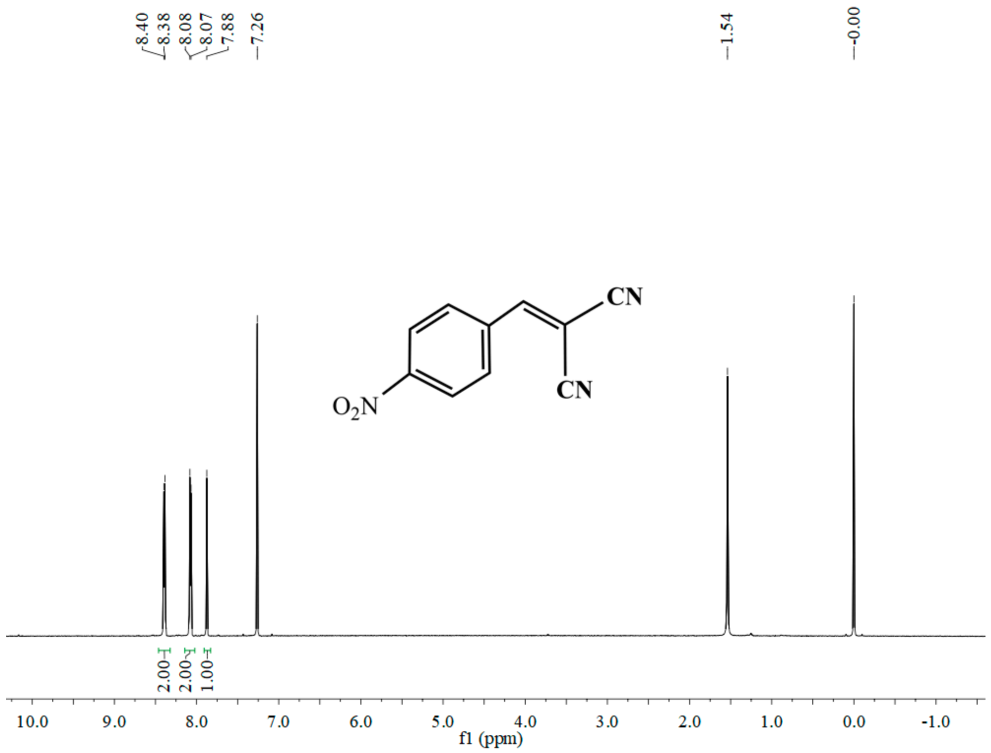

Figure S25 ^{1}H NMR of 2-(4-nitrobenzylidene)malononitrile.

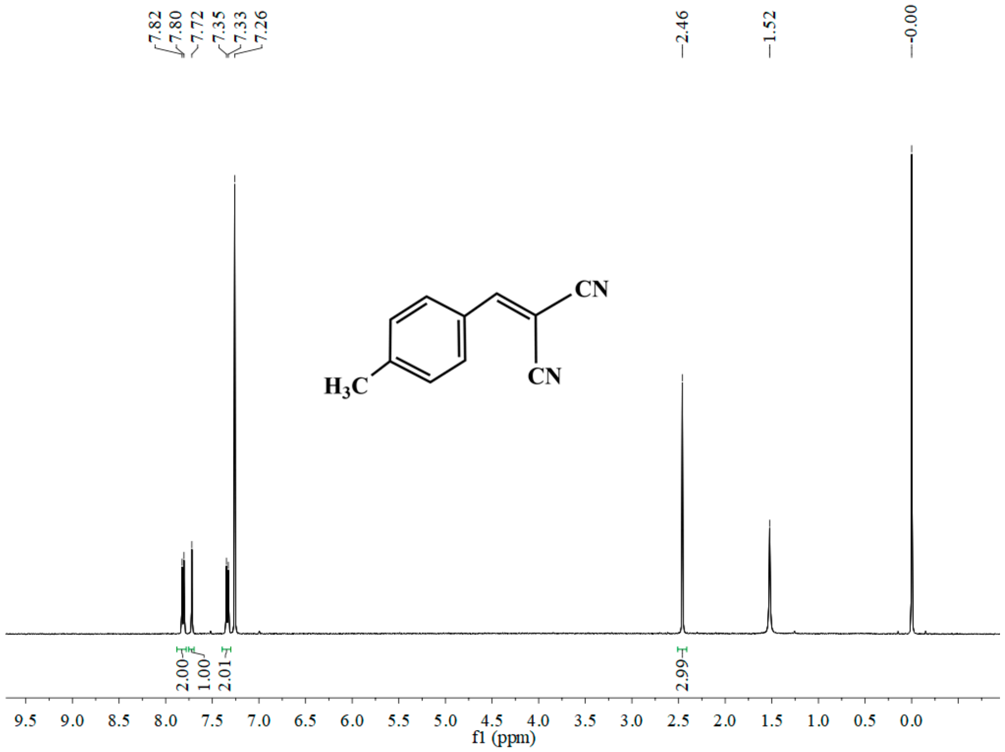

Figure S26 ^{1}H NMR of 2-(4-methylbenzylidene)malononitrile.

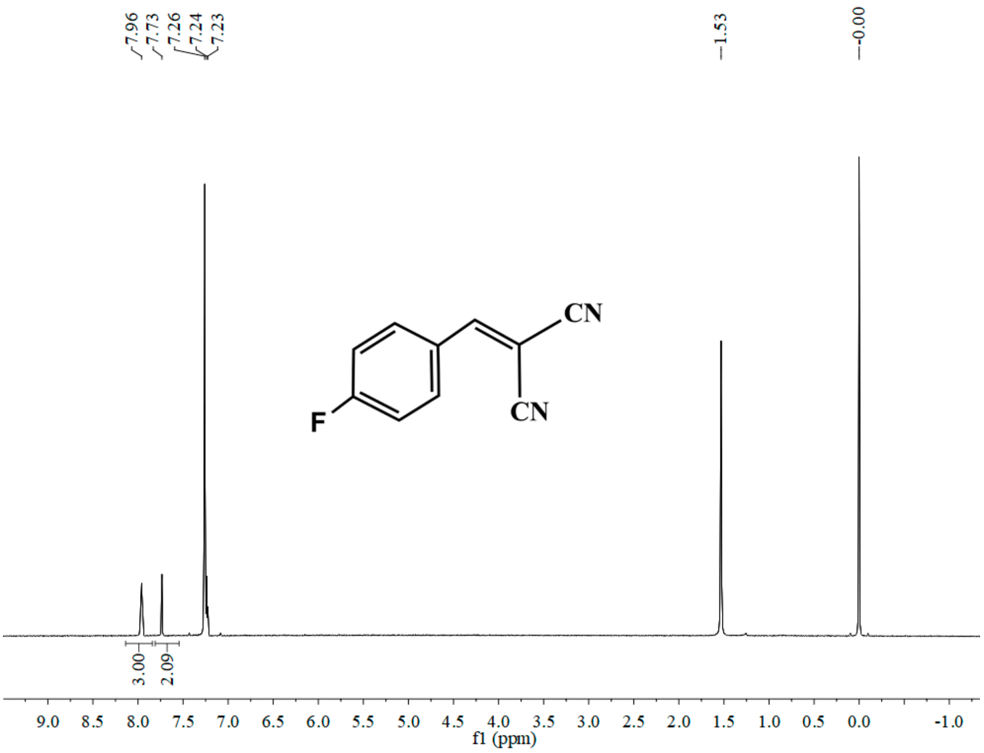

Figure S27 ^{1}H NMR of 2-(4-fluorobenzylidene)malononitrile.

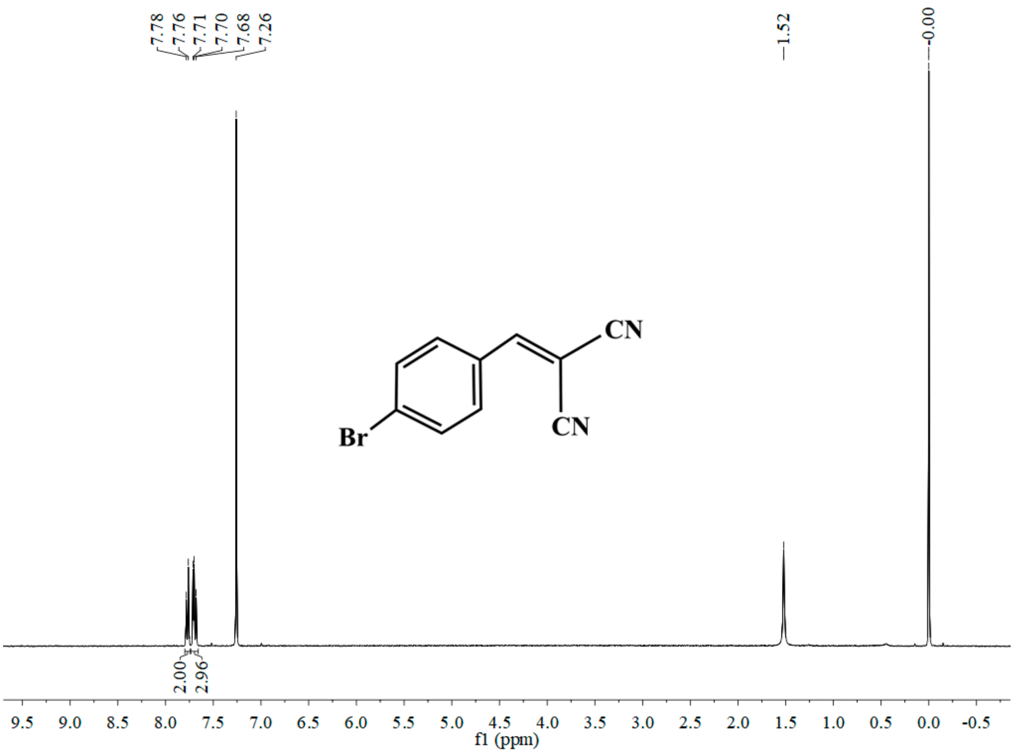

Figure S28 ^{1}H NMR of 2-(4-bromobenzylidene)malononitrile.

3. Tables S1-S2

Table S1 Surface chemical composition of the pristine and functionalized H-UiO-66-$(OH)_2$ (in at.%).

MOF	Zr	C	O	N	Si
H-UiO-66-$(OH)_2$	1.24	84.43	14.34		
H-UiO-66-$(OAPTMS)_2$	1.20	58.62	15.47	22.14	2.57
H-UiO-66-$(OAEAPTMS)_2$	1.26	57.13	20.06	16.33	5.21
H-UiO-66-$(OAEAEAPTMS)_2$	1.72	58.11	20.82	15.09	4.26

Table S2 Reaction of benzaldehyde and malononitrile under different catalyst conditions.

Entry	Catalyst	GC Yield (%)
1	No	3
2	H-UiO-66-$(OAPTMS)_2$	80
3	H-UiO-66-$(OAEAPTMS)_2$	85
4	H-UiO-66-$(OAEAEAPTMS)_2$	93

Table S3. Recyclability data of reaction of aldehydes and malononitrile under the same conditions.

**Entry**	**Product**	**Cycle 1 (%)**	**Cycle 2 (%)**	**Cycle 3 (%)**
1	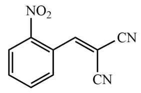	99	98	98
2	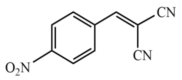	99	98	98
3	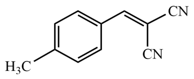	98	97	96
4	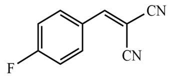	64	62	61
5	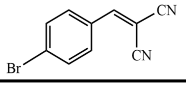	74	72	72
